# Sex-based differences in the gut-metabolism axis in aging and *Nrf2*-deficient mice

**DOI:** 10.1371/journal.pone.0357015

**Published:** 2026-09-21

**Authors:** Si Yang, Jiayi Zhou, Yuhan Yang, Pei Xu, Bolan Yu

**Affiliations:** 1 Department of Obstetrics and Gynecology, the Third Affiliated Hospital, Guangzhou Medical University, Guangzhou, China; 2 Guangdong Provincial Key Laboratory of Major Obstetric Diseases; Guangdong Provincial Clinical Research Center for Obstetrics and Gynecology; Guangdong-Hong Kong-Macao Greater Bay Area Higher Education Joint Laboratory of Maternal-Fetal Medicine, the Third Affiliated Hospital, Guangzhou Medical University, Guangzhou, China; 3 BioResource Research Center, the Third Affiliated Hospital of Guangzhou Medical University, Guangzhou, China; 4 Department of Gynecology, Foshan Women and Children Hospital Affiliated to Guangdong Medical University, Foshan, China; Emory University School of Medicine, UNITED STATES OF AMERICA

## Abstract

Hallmarks of aging are highly variable among individuals, and sexual dimorphism may contribute to these differences. However, the details are unclear.The gut-metabolism axisin naturally aging wild-type and *Nrf2* knockout (KO) mouse models was investigated. In this study, intraperitoneal glucose tolerance tests were performed on 12- and 48-week-old wild-type and *Nrf2* KO mice to assess glucose tolerance. Serum total cholesterol, triglyceride, low-density lipoprotein, and high-density lipoprotein levels were measured using biochemicaltest kits. The estradiol and testosterone levels in the serum were detected using enzyme-linked immunosorbent assays. Liver and adipose tissue morphology were examined using hematoxylin and eosin staining, and gut microbiota composition was analyzed using 16S rDNA sequencing of fecal samples.The results showed that glucose tolerance was significantly impaired in aging male *Nrf2*KO mice, and lipid accumulation in hepatic and adipose tissues increased in aging wild-type (WT) miceandaging *Nrf2*KO mice of both sexescompared with that in their young counterparts.Aged miceand *Nrf2* KO mice exhibited divergent responses in their serum lipid profiles.Notably, female *Nrf2* KO mice showed a significantly lowerα-diversity in gut bacterial composition, compared with both male *Nrf2* KO mice and female WT controls.In conclusion, the resultsindicatethat aging and *Nrf2* deficiency promoted weight gain and tissue lipid accumulation in both sexes but elicited sex-dimorphic differences in blood glucose stability, microbiota composition, and the magnitude of adiposity. Therefore, sex is an important factor contributing to age-related variations in gut microbiota and metabolism.

## Introduction

Aging is characterized by a gradual decline in homeostasis, and the major markers associated with this process include genetic and epigenetic alterations, dysregulation of nutrient sensing, organelle dysfunction, cellular senescence, and chronic inflammation [1]. One of the most accepted theories of aging, the “free radical theory of aging,” hypothesizes that the deterioration of tissues or organs during aging is due to excessive accumulation of reactive oxygen species [2]. NRF2(Nuclear factor erythroid 2-related factor 2)is a critical factor in the regulation of oxidative homeostasis, protecting tissues from free radical damage during aging. NRF2 binds to antioxidant response elements in promoter regions to regulate the expression of genes involved in antioxidant defense and detoxification. Thus, the onset and progression of age-related diseasesmay be inhibited byNRF2 [3,4]. Moreover, emerging evidence indicates that NRF2regulates lipid synthesis and glucose metabolism, which indirectly influences the metabolic axis during aging [5–7]. Therefore, NRF2 represents one of the most important targets for the anti-aging interventions.

Recent studies have shown that changes in the gut microbiota during agingmay contribute tothe development of age-related diseases like diabetes, Alzheimer’s disease, and Parkinson’s disease [8–10].Depletion in diverse microbiota species and microflora aberration,characterized by an increased *Proteobacteria* and decreased *Bifidobacterium*,arerelated to aging [11–15]. Variations in the *Firmicutes*-to-*Bacteroidetes* ratio are also frequently observed in older adults, including decreased *Firmicutes* and increased *Bacteroidetes*, and a reduced *Firmicutes*/*Bacteroidetes* ratio is potential predictive marker for aging [16,17]. A recent analysis revealed that centenarians harbor a higher abundance of gut microbiota dedicated to sustaining normal immune and metabolic functions, compared tothecontrol age group [18].

Aging is influenced by a complex interplay of genetic, dietary, lifestyle, and environmental factors [1]. While aging is often discussed collectively for both sexes, emerging evidence highlights significant differences driven by sex-specific biological divergence [19,20].Epidemiologically, women exhibit extended longevity with a lower prevalence of cardiovascular diseases during their premenopausal years. However, this advantage is lost after menopause, where their health status can decline, in some cases falling below that of age-matched men [21–23]. This sexual dimorphism isclearly reflected in lipid metabolism: middle-aged women typically have lower total cholesterol (TC), low-density lipoproteincholesterol(LDL-C), and non-high-density lipoprotein-cholesterol(HDL-C) concentrations than their male counterparts, whereas postmenopausal women exhibit elevatedtriglyceride (TG) and apolipoprotein B [24]. Given that both lipid metabolism and gut microbiota are key hallmarks of aging, we propose that sex acts as a critical modulating factor. By influencing these interconnected systems, sex may ultimately shape the trajectory of aging and the susceptibility to age-related diseases.

In this study, we used age-matched cohorts of naturally aged C57BL/6J wild-type (WT) and *Nrf2* knockout (KO) mice to investigate sexual dimorphism in aging-related metabolic dysregulation and gut microbiota. Through comparative analysis of sex-stratified metabolic phenotypes and the intestinal microbiota composition, we aimed to delineate the interplay between aging, sex, and the *Nrf2* gene, ultimately informing future anti-aging strategies.

## Materials and methods

### Animals

All experimental procedures were approved by the EthicsCommittee of the Third Affiliated Hospital of GuangzhouMedical University (approval number 2018[026]). Animal experiments were conducted in accordance with the National Institutes of Health Guide for the Care and Use of Laboratory Animals, and all efforts were made to minimize animal suffering and distress.

Specific pathogen-free, 8-week-old, WT C57BL/6J mice were purchased from the Guangdong Medical Laboratory Animal Center (Guangzhou, China).*Nrf2* KO mice were used as described in our previous study [25]. Mice were divided into eight groups: Mice were divided into eight groups: 12-week-old female WT mice (WT_C_F, n = 15), 48-week-old female WT mice (WT_A_F, n = 16), 12-week-old female KO mice (KO_C_F, n = 25), 48-week-old female KO mice (KO_A_F, n = 29), 12-week-old male WT mice (WT_C_M, n = 17), 48-week-old male WT mice (WT_A_M, n = 24), 12-week-old male KO mice (KO_C_M, n = 13), and 48-week-old male KO mice (KO_A_M, n = 10). All animals were housed in the Laboratory Animal Center of South China Agricultural University under a 12-hour light/dark cycle with *ad libitum* access to food and water. Mouse wellbeing and survival was monitored daily, and signs of illness, including bad appearance of fur,weight loss, hunching and overall motility were assessed daily for each animal. No animals died before scheduled euthanasia for sampling.

A total of 85 adult female mice and 64 adult male mice underwent baseline body weight measurement and intraperitoneal glucose tolerance test (IPGTT). Among these animals, 24 adult female mice and 32 adult male mice were subsequently included for the collection of serum, liver tissue, and gut microbiota samples, while the remaining animals were allocated to other independent research projects. The entire experimental procedure spanned 10 days, and euthanasia for sample collection was pre-scheduled as a predefined experimental endpoint. For the euthanasia process, each mouse was placed in a sealed chamber and humanely euthanized via carbon dioxide inhalation. After about 3–5 minutes, death was verified by complete cessation of respiration and absence of reflex responses to external stimuli, terminal blood collection was immediately performed via cardiac puncture. Subsequently, the liver and visceral adipose tissue was harvested via dissection. As all manipulations were conducted on deceased animalsafter euthanasia, no additional anesthesia or analgesic administration was necessary.

### Intraperitoneal glucose tolerance tests

Mice were subjected to a 12-hour fasting period with water available *ad libitum*before theIPGTT. At 8:00 AM on the following morning, mice were injected with 50% glucose solution (2 g/kg, intraperitoneal injection). Blood glucose was measured at baseline and at 30, 60, 90, and 120 minutes after IPinjection. During the IPGTT, blood samples were obtained by tail vein puncture, and each blood collection was finished within 30 seconds to minimize animal distress. Upon completion, the IPGTT curves were constructed, and the area under the curves (AUC) werecalculated.

### Sample collection

**Fig 1:**
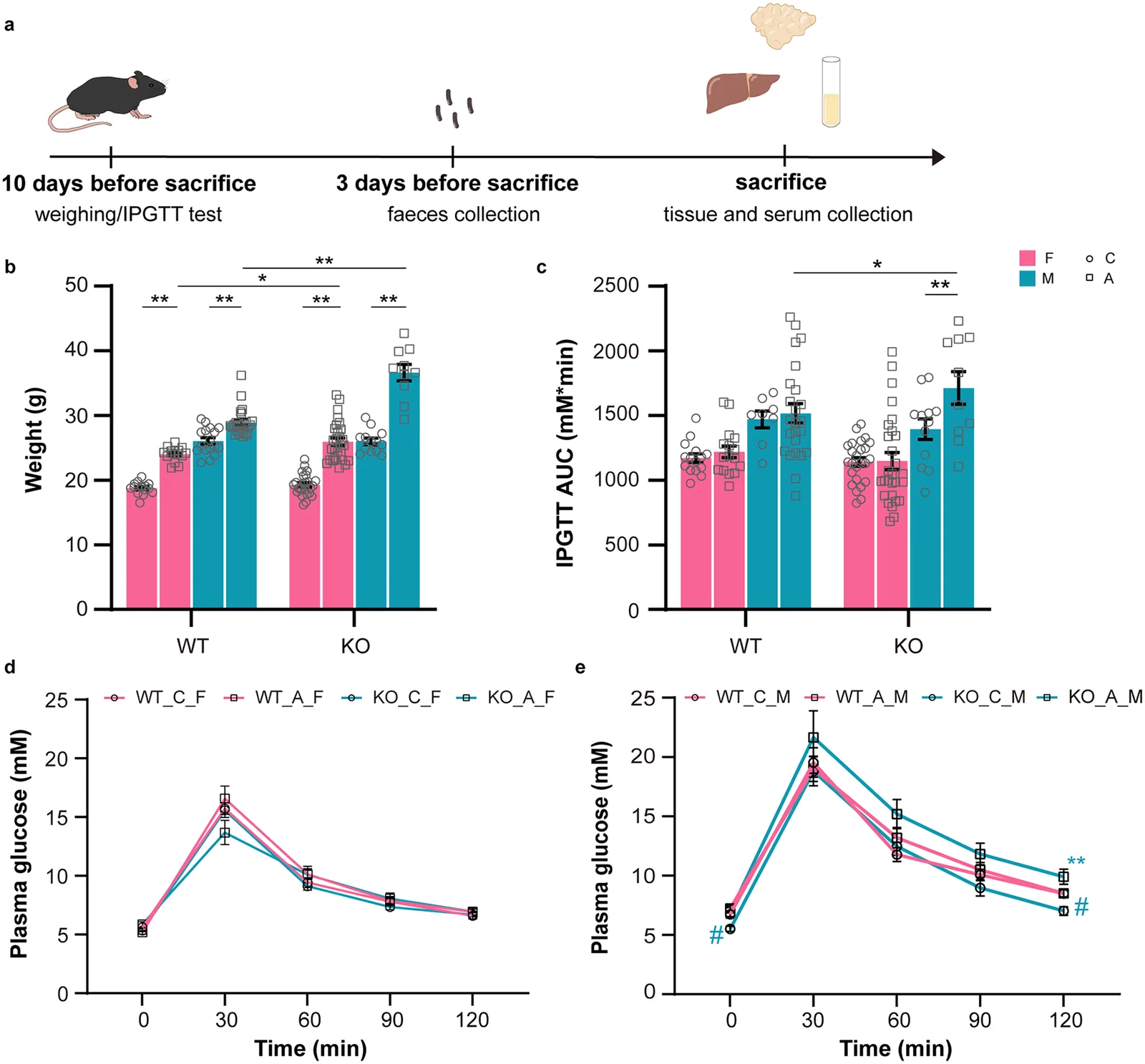
Effects of aging and *Nrf2*KO on body weight and blood glucose in mice. (a) The timeline for testing and sampling in mice. (b) Body weight of miceat experimental endpoint.(c) Area under the IPGTT curve in all mice groups. (d) Blood glucose variation during IPGTT test in femalemice. (e) Blood glucose variation during IPGTT test in malemice.Data are presented as mean ± SEM. (b and c) Data were analyzed usingtwo-way ANOVA with post hoc Tukey’s test, n = 10-29 per group, ^*^P < 0.05 and^**^P < 0.01.(d and e) Data were analyzed using two-way ANOVA with repeated measure method, followed by Tukey’s test for two-by-two comparisons,n = 8-28 per group,^**^P < 0.01 vs. young mice of the same genotype,^#^*P <* 0.05 vs. WT mice of the same age. Mouse groups include WT: wild-type; KO: *Nrf2* knockout; F: female; M: male; C: 12-week-old; and A: 48-week-old.

Mice were weighed and subjected to individual IPGTTs. One week later,fresh feces were collected. Three days after fecal collection, blood, liver and visceral adipose tissue samples were harvested via dissection (Fig 1a). Blood samples were left at room temperature for 1 hourand then centrifuged at 3000 rpm for 30 minutes at 4°C to separate the serum. All samples were stored at −80°C until analysis.

### Measurement of serum lipid and sex hormone levels

TG, TC, LDL, and HDL levels in peripheral serum were assessed using mouse TG, TC, LDL, and HDLassaykits (Cat#: A 110−1, A111-1, A112-1, A113-1,Jiancheng Bioengineering Institute, Nanjing, China) according to the manufacturer’s instructions. Estradiol (E2) in female mice and testosterone in male mice were measured using mouse E2and testosterone enzyme-linked immunosorbent assay (ELISA)kits (Enzyme-linked Biotechnology, Shanghai, China), respectively.

### Hematoxylin and Eosin (HE) Staining

Mouse liver tissue and abdominal fat were carefully separated through blunt dissection after euthanasia. Tissues were fixed in 10% neutral buffered formalin (Wexis, Guangzhou, China) for 6 hours, then dehydratedin an automated tissue dehydrator (Thermo Scientific Excelsior AS, MA, USA) and embedded in paraffin. After slicing (3µm thick) and dewaxing, sections were stained in hematoxylin for 1 minute.After a brief differentiation in 1% hydrochloric acid-ethanol, sections were rinsed and counterstainedwith eosin for 2 minutes. Histopathological changes in the liver were observed under a light microscope (Leica DM2500 LED,Hessen, Germany) at 400 × magnification. The number of hepatic fat vacuoles and the size of adipose tissue cells werequantifiedusing ImageJ software(NIH, Maryland, USA).

### Gut microbiota analysis

The total fecal microbial DNA was obtained through the Fecal Genome DNA Extraction Kit (AU46111-96, BioTeke, China) according to the manufacturer’s instruction manual. The DNA was quantified by Qubit (Invitrogen, USA), and the variable region (V3-V4) of 16S rDNA was amplified via PCR with the following primer sequences: 341F (5′-CCTACGGGNGGCWGCAG-3′) and 805R (5′-GACTACHVGGGTATCTAATCC-3′). The PCR product was purified using AMPure XT Beads (Beckman Coulter Genomics, Danvers, MA, USA) and quantified using Qubit (Invitrogen, USA). The amplicon pools were prepared for sequencing and the size and quantity of the amplicon library were assessed on Agilent 2100 Bioanalyzer (Agilent, USA) and with the Library Quantification Kit for Illumina (Kapa Biosciences, Woburn, MA, USA), respectively. The libraries were sequenced on NovaSeq PE250 platform. The DNA extraction, PCR and library preparation were performed by LC-Bio Technology Co., Ltd., Hangzhou, China.

Paired-end reads were merged based on overlap. Quality control and chimeric removal were performed using fqtrim (v 0.94) and Vsearch (v 2.3.4) software. After obtaining the final characteristic sequences, diversity analysis, species taxonomic annotations, and differential analysis were performed. Species annotation was performed based on the ASV sequence file using the SILVA database (Release 138, https://www.arb-silva.de/documentation/release138/, annotation threshold: --min_confidence 0.7) and the NT-16S database (Release 20230718, annotation thresholds: --min_ident 90 --min_cov 80 --max_e 1e-5), and statistically analyze the abundance of species at each taxonomic level in each sample according to the ASV abundance table. The SILVA databaseprovides taxonomic information down to the genus level. The supplementary annotation using the NT-16S database is mainly to add species-level taxonomic details. Results were generated on the LC-Bio OmicStudio platform (https://www.omicstudio.cn). Linear discriminant analysis (LDA) was carried out identifying microbial taxa with significant differences between groups.

### Statistical analysis

Data were analyzed using GraphPad Prism 9.0 (GraphPad, California, USA). Multiple groups were compared using two-way ANOVA or two-way ANOVA with repeated measures followed by Tukey’s tests for two-by-two comparisons. Characteristic microbial populations were compared using Kruskal-Wallis rank sum tests. The Wilcoxon rank sum test was applied to determine whether all subspecies in differential species converged at the same taxonomic classification level.The 16S rDNA sequencing results were visualized in R (The R Foundation, Vienna, Austria), differences of P value ≤ 0.05 were considered significant.

## Results

### Aging and *Nrf2*KO contributed to weight gain in male and female mice

Given that sex differences in body weight and blood biochemistry have been extensively studied, our study majorly focused on whether the changes induced by age and genotype are different between female and male mice. Longitudinal analysis revealed significant age-related weight gain in both male and female mice by 48weeks, an effect that was exacerbated by*Nrf2* deficiencyin an age-and sex-dependent manner (Fig 1b). The greatest increase occurred in aged*Nrf2*KO males (KO_A_M), whose weight gain was 3.70-fold higher than that ofaged WT males(10.62g vs. 2.88g, compared with their young counterparts). Female mice showed modest weightgains,with KO_A_F mice gaining only 1.27-fold more weight than WT_A_F mice (6.68g vs. 5.28g, compared with their young counterparts). Notably, body weights did not differ between genotypes at 12 weeks in either sex, indicating the metabolic consequences of *Nrf2* deficiency are cumulative and emerge with advancing age.

### *Nrf2*KO may induce dysglycemia in control and aged male mice

Based on IPGTTs, blood glucose betweenWT control and aged mice did not differ in either sex (Fig 1c–1e). However, *Nrf2* ablation unmasked a striking sex-dependent phenotype. Control*Nrf2*KO males (KO_C_M) exhibited reduced fasting and post-challenge blood glucose levels relative to WT controls (Fig 1e), indicative ofcompensatory insulin sensitization. This phenotype was reversed with aging;aged *Nrf2*KO males (KO_A_M)displayed sustained hyperglycemia across all IPGTT timepoints(120 min: 9.84 ± 0.58 vs. KO_C_M 7.03 ± 0.37 mM, *P <* 0.01; Fig 1e). Consistently, the IPGTT AUC was significantly higher in KO_A_M mice than in KO_C_M (*P <* 0.01), and WT_A_M mice (*P <* 0.05; Fig 1c). These findings delineate an*Nrf2*-dependent sexual dichotomy in glucoregulatory adaptation, where youthful compensation transitions to age-related dyshomeostasis in males.

### Effects of aging and *Nrf2*KO on blood lipid profiles in male and female mice

**Fig 2:**
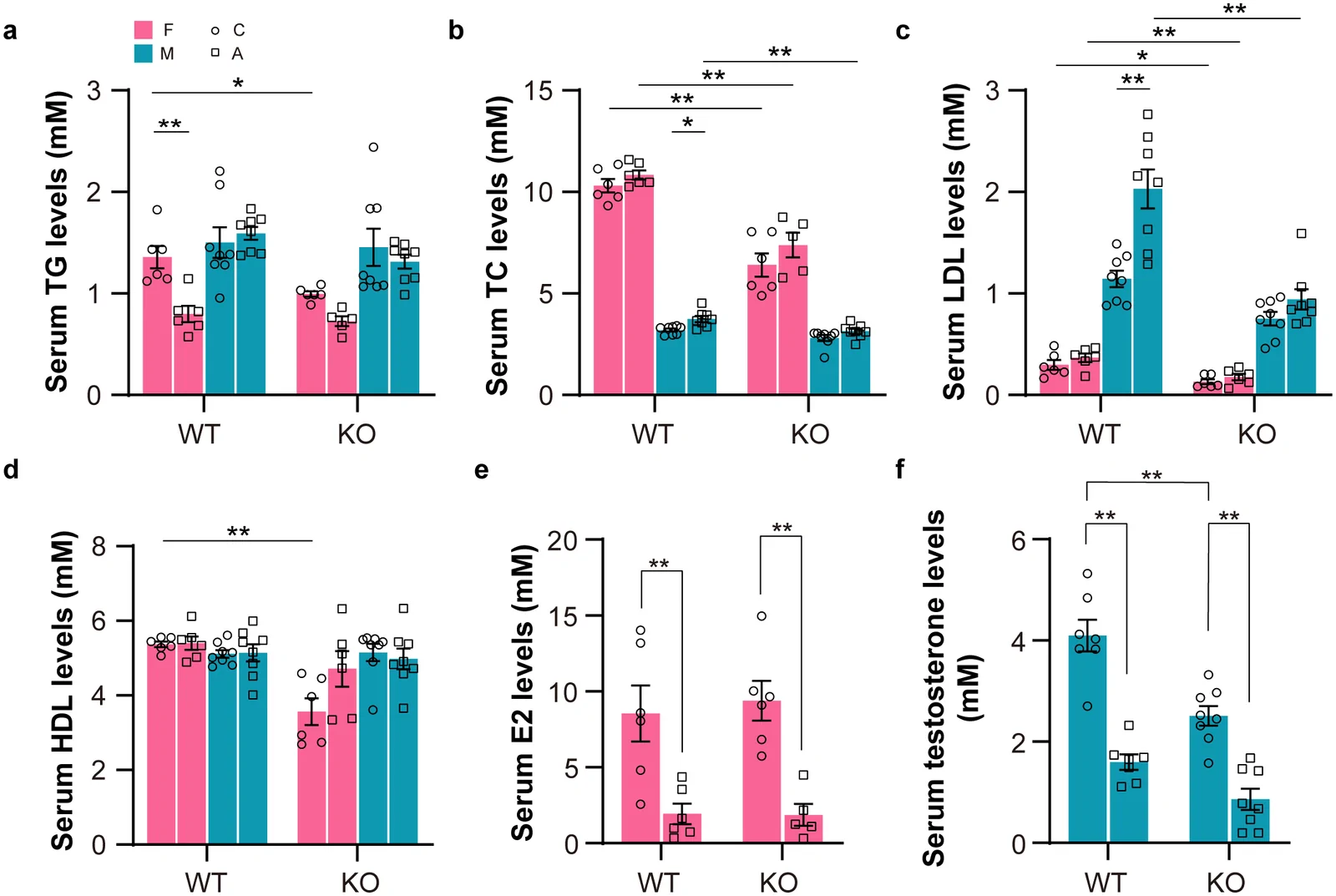
Aging and *Nrf2*KO disrupted the homeostasis of serum lipids and sex hormones in mice. (a-d) Serum levels of TG, TC, LDL, and HDL in mice. (e) Serum estradiol (E2) levels in females. (f) Serum testosterone levels in males. Bar graphs show means ± SEM, n = 6-8 per group,^*^P < 0.05 and ^**^P < 0.01 by two-way ANOVA with post hoc Tukey’s test. Mouse groups includeWT: wild-type; KO: *Nrf2* knockout; F: female; M: male; C: 12-week-old; and A: 48-week-old.

Lipidomic profiling revealed sex-specific metabolic patterns during aging and *Nrf2* depletion. In WT cohorts, TG was significantly reduced in aged female micecompared to young controls (WT_A_F vs. WT_C_F: *P <* 0.01; Fig 2a), but not in males. Both sexes exhibited age-related dyslipidemia characterized by elevated TC and LDL, though the magnitude was greater inmales (TC: *P <* 0.05; LDL: *P <* 0.01 vs. WT_C_M) than in females(TC: *P >* 0.1; LDL: *P >* 0.1 vs. WT_C_F; Fig 2b and 2c).

*Nrf2*knockoutremodeledlipid profiles. *Nrf2*KO mice displayed decreased serum TG (KO_C_Fvs WT_C_F: *P <* 0.05), TC (KO_C_F vs WT_C_F:*P <* 0.01, KO_A_F vs WT_A_F: *P <* 0.01, KO_A_M vs WT_A_M: *P <* 0.01), and LDL levels (KO_C_F vs WT_C_F: *P <* 0.05; KO_A_Fvs WT_A_F: *P <* 0.01, KO_A_M vs WT_A_M: *P <* 0.01) compared with WT mice (Fig 2b and 2c). Notably, the effect on HDL was sex-specific, with significantly greater suppression in females than in male mice(WT_C_F vs. KO_C_F:*P <* 0.01; Fig 2d).

### Effects of aging and *Nrf2*KO on serum sex hormone levels in male and female mice

Comparative analysis of serum sex hormones revealed distinct age-relatedendocrine patterns in males, and *Nrf2*-dependent endocrine patternsin females.In WT mice, serum E2 levels were reduced by 77% in aged femalescompared with younger females(WT_A_FvsWT_C_F:*P <* 0.01; Fig 2e), and testosterone levels were 61% lower in aged males than in younger males (WT_C_Mvs. WT_A_M:*P <* 0.01; Fig 2f). *Nrf2* ablation induced sexually dimorphic hormonal responses. KO males exhibited decreasedtestosterone (KO_C_M vs. WT_C_M: *P <* 0.01),while females maintained stable circulating E2 levels(P = 0.96).

### Aging and *Nrf2*KO aggravated hepatic lipid accumulation in male and female mice

**Fig 3:**
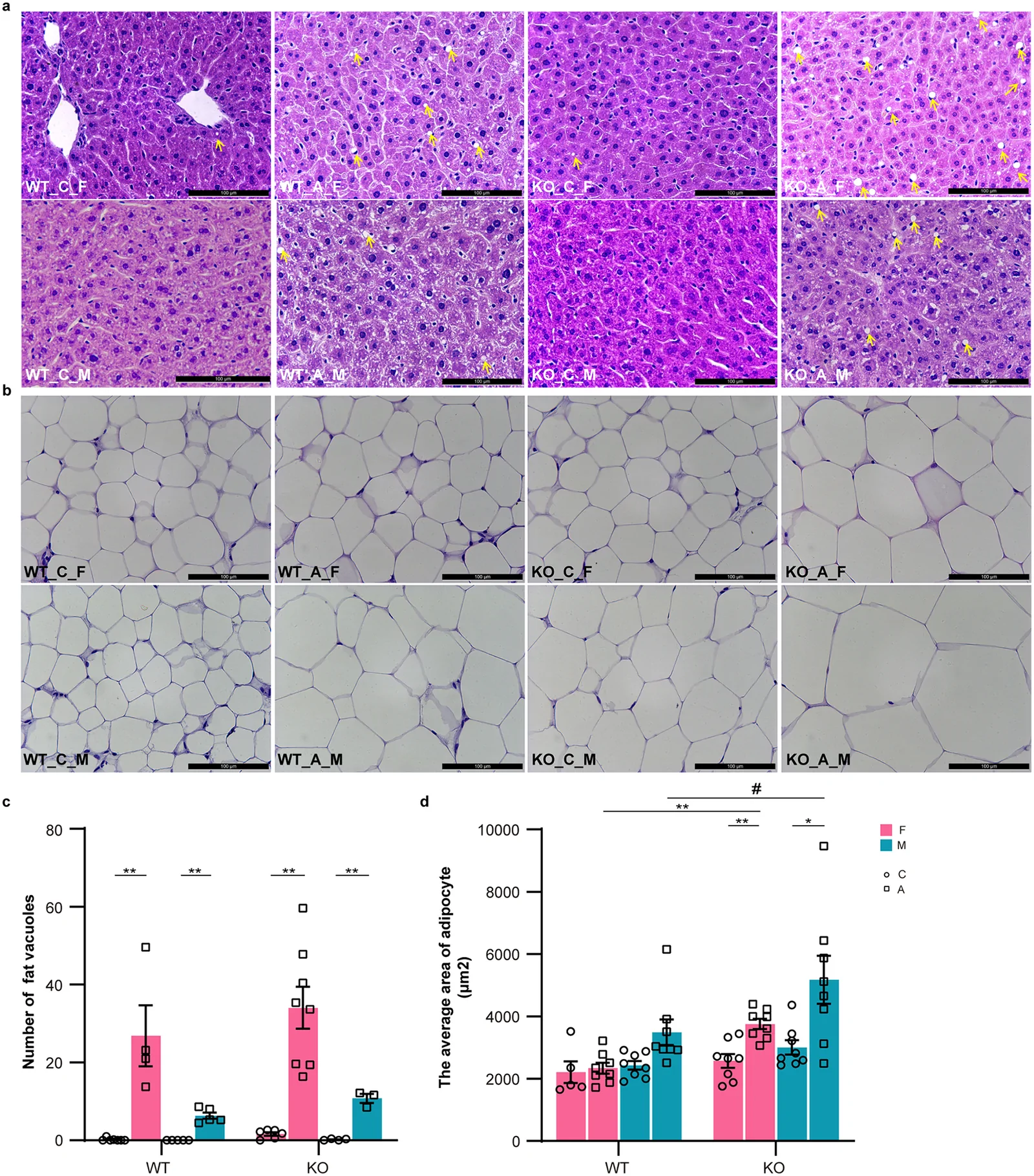
Aging and *Nrf2*KO promoted the accumulation of fat vacuoles in the liver and induced adipocyte hypertrophy in mice adipose tissue. (a) HE-stainedliver sections from mice. Yellow arrows: lipid vacuoles. (b) HE-stainedadipose tissuesections from males. (a and b) 10 × 40 magnification. (c) Number of fat vacuoles in the liver of mice. (d) Average adipocytes areas in mice. (c and d) Bar graphs show means ± SEM,n = 6-8 per group,^#^*P* < 0.1, ^*^P < 0.05 and^**^P < 0.01 by two-way ANOVA with post hoc Tukey’s test. Mouse groups includeWT: wild-type; KO: *Nrf2* knockout; F: female; M: male; C: 12-week-old; and A: 48-week-old.

The liver and adipose tissue are critical sitesfor lipid metabolism and energy storage and are significantly affected by oxidative damage [26,27]. Thus, these organs are suitable targets for assessing oxidative damage during aging. HE-stained tissues were analyzed to assess the influence of aging and *Nrf2* deficiency on liver metabolism. Substantial age-dependent lipid depositionsweredetected in WT mice. Hepatic fat vacuole counts were significantly higher in male and female aged mice than in their younger counterparts(WT_C_F vs. WT_A_F, WT_C_M vs. WT_A_M: *P <* 0.01, Fig 3a and 3c). Strikingly, genetic ablation of *Nrf2* exacerbated this phenotype. Aged*Nrf2*KOfemales exhibiteda greater increase in fat vacuolesthan males(compared with WT aged mice), with females gaining 7.14 additional vacuoles on average versus 4.46 in males.

### Aging and *Nrf2*KO increased adipocyte area in male and female mice

Adipose tissue, as a crucial site for lipid storage, is significantly influenced by aging and *Nrf2* deficiency. Histomorphometric analysis of HE-stained sections revealed age-related adipocyte remodeling, characterized by significant cellular hypertrophy in both sexes compared with younger mice (Fig 3b and 3d). Consistent with the findings in the liver, *Nrf2* ablation exacerbated age-associated adipocyte expansionin a sex-dependent manner, with female KO mice exhibiting a more pronounced increase (KO_A_Fvs WT_A_F: *P <* 0.01, KO_A_M vs WT_A_M: *P <* 0.1, Fig 3d).

### Effects of aging and *Nrf2*KO on intestinal microbiotadiversity in male and female mice

**Fig 4:**
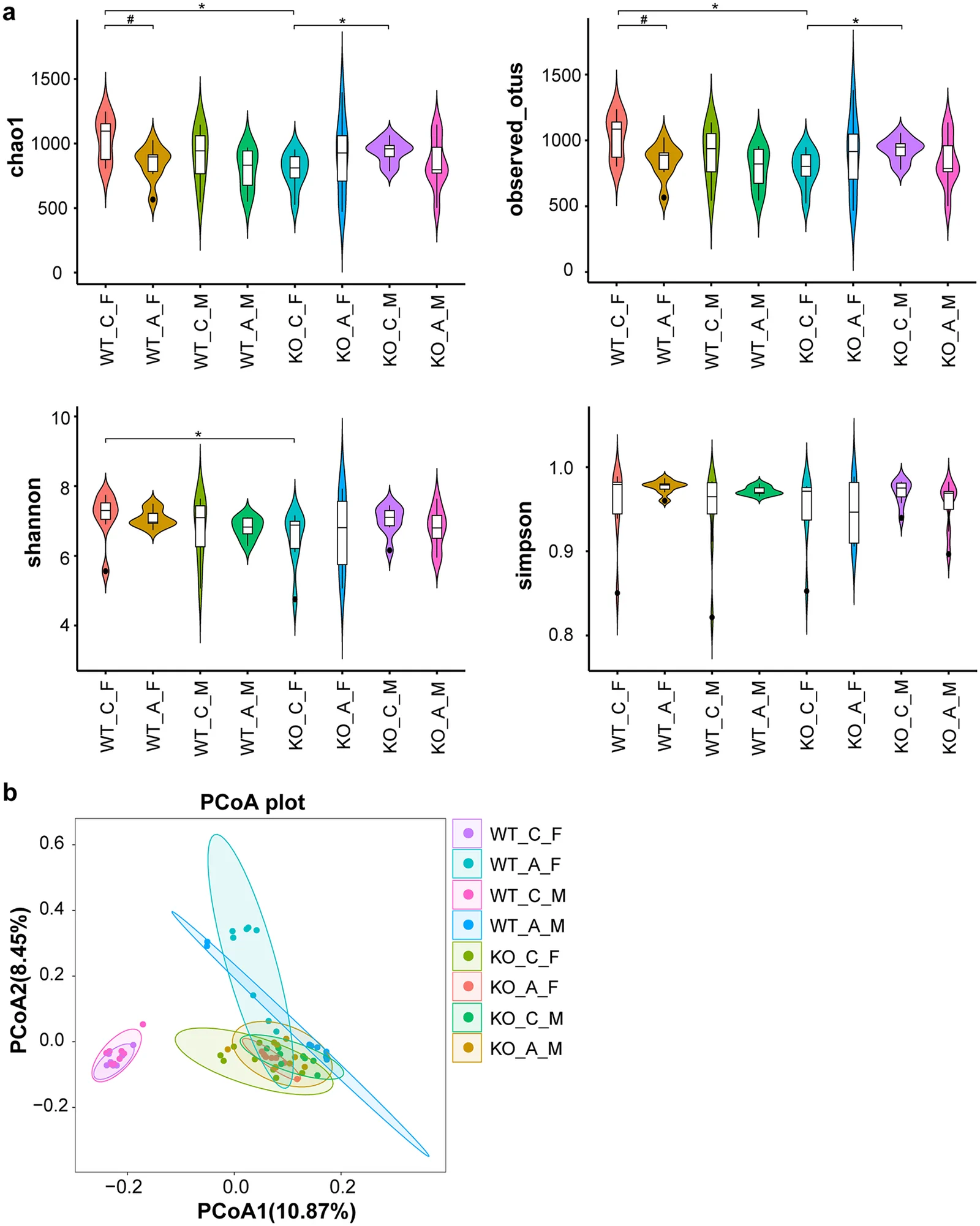
Effects of aging and *Nrf2*KO on mouse gut microbiota diversity and structure. (a)Violin plots of OTUs, Chao1, Shannon and Simpson indices of mice, n = 6-8 per group,^#^*P* < 0.1 and ^*^P < 0.05 by Kruskal-Wallis rank sum test. (b) PCoA of gut microbiota composition, *P* = 0.001 by ANOSIM (Analysis of similarities)of PCoAanalysis. Mouse groups includeWT: wild-type; KO: *Nrf2* knockout; F: female; M: male; C: 12-week-old; and A: 48-week-old.

Microbial α-diversity declined with aging in WT mice of both sexes.Aged WT mice exhibited lower Chao1, OTUs, and Shannon indices compared to younger controls (Chao1, OTUs: WT_C_F vs. WT_A_F: *P <* 0.1; Fig 4a). However,*Nrf2*genetic ablation induced sexually dimorphic variations. In females, Chao1, OTUs, and Shannon indices decreased inyoung KO mice (KO_C_Fvs WT_C_F: *P <* 0.05), while Shannon and Simpson indices were slightly reduced in aged KO mice compared with WT cohorts (Fig 4a). In contrast, no significant changes were detectedamong male KOmice. Cross-sex comparative analysis revealed that younger WT females (WT_C_F) also exhibited higher baseline α-diversity and greaterα-diversity loss upon*Nrf2* deletionthan their male counterparts.KO_C_F mice displayed lower Chao1 (*P <* 0.05), OTUs (*P <* 0.05), Shannon, and Simpson indices compared to KO_C_Mmice (Fig 4a).

Unweighedprincipal coordinate analysis (PCoA) was conducted to identify differences in the composition of intestinal microbiota. As shown in Fig 4b, the WT_C_F and WT_C_M groups were closely positioned on the coordinate axes and far from the other groups, while the WT_A_F, WT_A_M, KO_C_F, KO_C_M, KO_A_F, and KO_A_M groupswere clustered, suggesting that the intestinal microbiota structures of aged and *Nrf2*KOmicewere similar in both sexes.

### Effects of aging and *Nrf2*KO on the composition of intestinal microbiota in male and female mice

**Fig 5:**
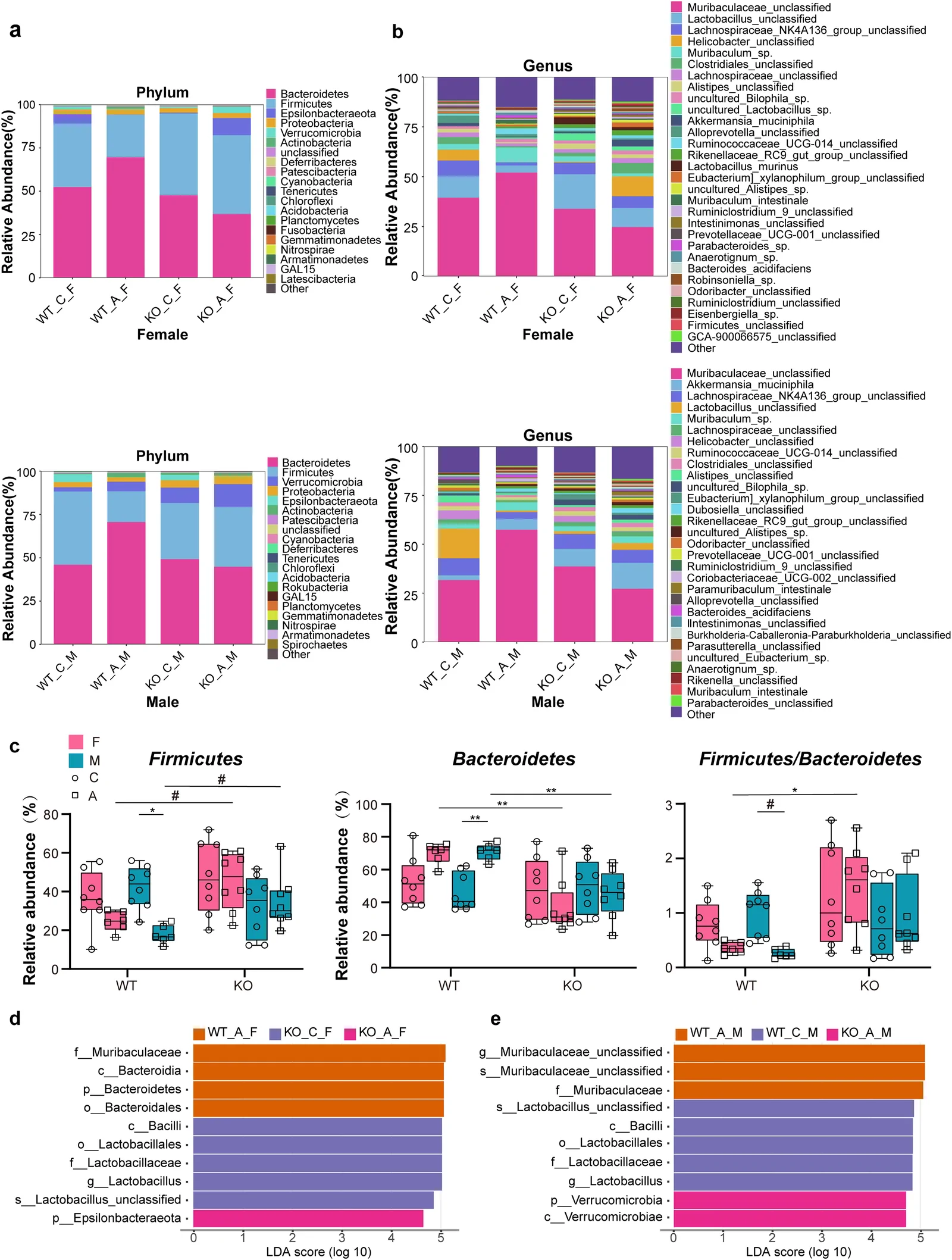
Aging and *Nrf2*KO altered gut microbiota composition in different mouse groups. (a) Phylum-level taxonomic profile in female and male mice.(b) Genus-level taxonomic profiles in female and male mice. (c) Relative abundance of *Firmicutes*, Bacter*oidetes*, and *Firmicutes*/*Bacteroidetes* ratio in mice (from the left to the right). Data was presented as quartiles, n = 6-8 per group,^#^*P* < 0.1, ^*^P < 0.05 and ^**^P < 0.01 by two-way ANOVA with post hoc Tukey’s test. (d) LEfSe analysis of differentially enriched taxa in females. (e) LEfSe analysis of differentially enriched taxa in males. (d, e) Only the top 10 dominant bacterial taxa in all groups, along with the group that exhibited the highest relative abundance of each taxon, are shown.Mouse groups includeWT: wild-type; KO: *Nrf2* knockout; F: female; M: male; C: 12-week-old; and A: 48-week-old.

To analyze the intestinal microbiota composition in mice, the top 20 and top 30 most abundant species were selected at the phylum and genus levels, respectively (Fig 5a and 5b). At the phylum level, *Bacteroidetes* and *Firmicutes*predominated (Fig 5a). The abundance of *Bacteroidetes* increased (WT_A_Mvs WT_C_M: *P <* 0.01) while*Firmicutes* decreased (WT_A_M vs WT_C_M: *P* < 0.05) in aged mice compared with control mice, leading to a reduced *Firmicutes* to *Bacteroidetes*(*F/B*) ratio(WT_A_M vs WT_C_M: *P <* 0.1; Fig 5a and 5c). *Nrf2*KO induced a marked decrease in *Bacteroidetes* abundanceand an increase in *Firmicutes* abundance inyoung females and aged males and females, resulting in a significant increase in *F/B* ratio(*Bacteroidetes*: KO_A_Fvs WT_A_F: *P <* 0.01, KO_A_M vs WT_A_M: *P <* 0.01; *Firmicutes*: KO_A_F vs WT_A_F: *P <* 0.1, KO_A_M vs WT_A_M: *P <* 0.1; F/B: KO_A_F vs WT_A_F: *P* < 0.05; Fig 5a and 5c).

Sexual dimorphism was also observed among additional microbiota phyla.In female mice, the relative abundance of *Proteobacteria* was uniform among groups, and the abundance of *Epsilonbacteraeota* and *Verrucomicrobia*wereincreased in the aged*Nrf2* KO females compared to the aged WT counterparts (Fig 5a). In male mice, the proportion of *Verrucomicrobia* and *Proteobacteria* increasedin *Nrf2* KO mice compared to WT controls; however,*Epsilonbacteraeota* was rarely detectedboth inaged WT and *Nrf2*KOmice.

At the genus level, the gut microbiota exhibited substantial diversity, dominated by*Muribaculaceae_unclassified*, *Lactobacillus_unclassified*,*Akkermansia_muciniphila*, and *Lachnospiraceae_NK4A136_group_unclassified*(butyrate-producing bacterium),with group-specific abundance patterns (Fig 5b). Notably, the proportion of *Lachnospiraceae_NK4A136_group_unclassified*decreased in the aged WT mice compared to the young WT mice, but remained unchanged for *Nrf2* KO mice in both sexes (Fig 5b). In addition, sex-biased distribution was evident in*Akkermansia_muciniphila*, a biomarker of gut health [28,29], with significantly higher abundance in males than females.

Linear Discriminant Analysis Effect Size (LEfSe) differential analysis is a biomarker discovery method specifically designed for high-dimensional microbiome data, which integrates statistical significance testing with biological effect quantification to identify differential taxa that are both statistically significant and biologically influential across multiple sample groups. The LDA score is the core effect size metric in LEfSe, where a higher value indicates a stronger contribution of the taxon to group discrimination. In this study, LEfSe differential analysis identified sexually dimorphic discriminatory taxa with an LDA score > 2.0 across all comparison groups. Only the top 10 dominant bacterial taxa in all groups, along with the group that exhibited the highest relative abundance of each taxon, are presented in the results (Fig 5d and 5e). In female mice, the abundance of *Muribaculaceae*, *Bacteroidia*, *Bacteroidetes*, and *Bacteroidales*increased in the WT_A_F group. *Bacilli*, *Lactobacillales*, *Lactobacillaceae*, *Lactobacillus*, and *Lactobacillus_unclassified*were elevated in the KO_C_F group.Likewise, the abundance of *Epsilonbacteraeota*was higher in the KO_A_F group (Fig 5d). In males, the abundance of *Muribaculaceae_unclassified* and *Muribaculaceae*increased in the WT_A_M group. *Lactobacillus_unclassified*, *Bacilli*, *Lactobacillales*, *Lactobacillaceae,* and *Lactobacillus* were higher in the WT_C_M group. And the abundance of *Verrucomicrobia* and *Verrucomicrobiae*washigher inthe KO_A_M group (Fig 5e). Cross-group comparisons revealed that *Muribaculaceae*wasabundant in aged WT mice of both sexes, and the dominant microbiota in KO_C_F and WT_C_M groupsare highly similar (Fig 5d and 5e).

## Discussion

Although earlier investigations established sex-specific dimorphism in many diseases, including cardiovascular disease and diabetes [20], the effects of sex on aging are poorly understood. Our results revealed interesting sexual dimorphism in metabolic and gut microbiota responses to *Nrf2* deficiency during aging. Aged *Nrf2*-deficient males exhibited severe metabolic dysregulation, including exacerbated weight gain, glucose intolerance, tissue lipid accumulation, and testosterone depletion. In contrast,femalesmaintained glucose tolerance but displayed an unexpectedphenotype, including significantly reduced circulating lipidsand exacerbated hepatic lipid accumulation and adipocyte hypertrophy. Gut microbiota analysis revealed sex-stratified adaptations. Beneficial *Lachnospiraceae_NK4A136*was depleted in agedWT mice, while the *Firmicutes*/*Bacteroidetes*ratio was elevated in both agedWT and *Nrf2* KO mice. However, the potential pathogenic bacteria-*Proteobacteria*accumulatedand the mucosal health associated*Verrucomicrobia*increasedin aged and *Nrf2* KOmales. These divergent outcomes may be driven by estrogen-mediated protection in females versus testosterone suppression in males. The oxidative collapse in males suggests that *Nrf2* signaling intersects with sex-specific hormonal landscapes to dictate metabolic fate and microbiota dynamics.

Sexual dimorphism was evidentin the metabolic and microbial responsesto *Nrf2* deficiency. Aged *Nrf2* KO male mice exhibited a 3.70-fold greater weight gain than their WT counterparts, accompanied by profound glucose dysregulation. Females exhibited attenuated disturbances, with only a 1.27-fold weight gain and stable glucose tolerance. This divergence mirrors human epidemiological trends. Aging males exhibit higher diabetes susceptibility than females [30], suggesting conserved sex-specific regulatory mechanisms. This dichotomy signifies an estrogen-mediated protective mechanism against redox imbalance-induced dysregulation. Apreviousreport showedthat estrogen protects kidneys against redox imbalance [31].

*Nrf2* ablation elicited paradoxical changesin systemic lipid clearance and tissue-specific depositionin both sexes. In WT cohorts, TC and LDL were elevated, and lipid deposition was increased in both sexeswith aging (more pronounced in males).However, TG was significantly reduced in females compared with males, underscoring inherent sexual divergence in age-related lipid handling. Strikingly,*Nrf2* deficiency reversedthesechanges in circulatory lipids.Serum TG, TC, LDL, and HDL levels were significantly reduced, but hepatic lipid accumulation and adipocyte hypertrophy were paradoxically exacerbated in female*Nrf2*KO mice compared with WTcounterparts.Aged*Nrf2*KOmales displayedsimilar but attenuated circulating lipids and visceral adiposevariations compared to females,while demonstrating severe testosterone suppression.

Circulating lipids increased with fat accumulation in aged WT mice. *Nrf2*KO dissociated these effects: circulatory lipids were reduced, but abnormal fat deposition was increasedin both sexes. This suggests potentially impairing reverse cholesterol transport and trapping lipids in peripheral tissues. The sexual dichotomy may arise from disrupted hormonal-metabolic crosstalk in*Nrf2*KO mice. In females, stable estradiol levels failed to compensate for *Nrf2*-deficientcirculating adipose suppression. In contrast, testosterone was depleted in males, as a previous study showed that reduced testosterone in Nrf2/HO-1(heme oxygenase-1) axis impaired testicular torsion [32], creating a self-reinforcing cycle of oxidative stress and lipid peroxidation.

Dysbiosis is an additional hallmark of aging. The microbial community in the intestinal tract is highly variable among individuals due to host genetic variants, dietary factors, lifestyle habits, and environmental conditions [1]. Our results demonstrated that variations in gut microbiota may also be linked to sex-stratified responses to specific gene loss. Aged mice and *Nrf2* KO mice were separated from young WT mice in the PCoA of gut microbiota, suggesting that the loss of antioxidative capability represents senescent characteristics in microbiota homeostasis. Detailed analyses indicated functional divergence. The *Firmicutes*/*Bacteroidetes* ratio was elevated in male and female aged*Nrf2*KO mice (more pronounced in females), correlating with previously reported decreases in circulating lipids and enhanced visceral adiposity [33]. *Verrucomicrobia* and *Epsilonbacteraeota* were increased in aged*Nrf2*KOfemale mice, while*Verrucomicrobia* and *Proteobacteria*were more abundant in aged*Nrf2*KO males compared with their aged WT counterparts.*Proteobacteria*, and *Epsilonbacteraeota* are potential pathogenic bacteria [34,35], and their distribution differences may be related to the metabolic variation in males and females after *Nrf2*knockout. Microbiota in female mice exhibited heightened vulnerability to α-diversity loss, aligning with the sex-specific microbiota destabilization induced by metabolic stress in a previous study [36]. Conversely, males retained microbial diversity, but accumulated*Verrucomicrobia*potentially exacerbated their hyperglycemic phenotype [37]. In addition, butyrogenic *Lachnospiraceae_NK4A136*was depleted in both sexes, suggesting a universal impairment of gut barrier function, althoughmales may partially offsetthis dysfunction via a higher abundance of baseline *Akkermansia*. *Akkermansia is*a genus with anti-inflammatory properties that may partially mitigate intestinal barrier dysfunction [29], whichexplaining the preserved circulating lipid profiles and abnormal deposition after *Nrf2*knockout.

While our work reveals sex-specific interactions between *Nrf2* signaling and metabolic regulation during aging, this study has limitations that require consideration. Our analysis was restricted to 12-week-old (young adult, equivalent to ~20 years in humans) and 48-week-old (middle-aged, equivalent to ~40 years in humans) mice, and this time point may not be sufficient to capture robust aging-related alterations across all experimental groups. Further investigations in mice aged18 months or olderaretherefore required to fully elucidate the complexinteractions among aging, biological sex, and *Nrf2*genotype.

## Conclusion

In summary, this study demonstrated that aging and *Nrf2* deficiency promoted weight gain and tissue lipid accumulation in both sexes but elicited sex-dimorphic differences in blood glucose stability, gut microbiota composition, and the magnitude of adiposity. Therefore, sex may contribute to varied microbiota dynamics and metabolic homeostasis while aging with different genetic backgrounds. Our results provide the basis for future individualized aging and therapeutic interventions.

## Supporting information

S1 Fig HE-stained liver sections indifferentgroups (10 × 10 magnification). Mouse groups include WT: wild-type; KO: *Nrf2* knockout; F: female; M: male; C: 12-week-old; and A: 48-week-old.
(TIF)

S2 Fig HE-stained liver sections in differentgroups (whole-slide view). Mouse groups include WT: wild-type; KO: *Nrf2* knockout; F: female; M: male; C: 12-week-old; and A: 48-week-old.
(TIF)

S3 Fig HE-stained adipose tissue sections in differentgroups (10 × 10 magnification). Mouse groups include WT: wild-type; KO: *Nrf2* knockout; F: female; M: male; C: 12-week-old; and A: 48-week-old.
(TIF)

S4 Fig HE-stained adipose tissue sections in differentgroups (whole-slide view). Mouse groups include WT: wild-type; KO: *Nrf2* knockout; F: female; M: male; C: 12-week-old; and A: 48-week-old.
(TIF)
